# RETRACTED: Lai et al. MicroRNA-21 Plays Multiple Oncometabolic Roles in the Process of NAFLD-Related Hepatocellular Carcinoma via PI3K/AKT, TGF-β, and STAT3 Signaling. *Cancers* 2021, *13*, 940

**DOI:** 10.3390/cancers16081597

**Published:** 2024-04-22

**Authors:** Chi-Yu Lai, Kun-Yun Yeh, Chiu-Ya Lin, Yang-Wen Hsieh, Hsin-Hung Lai, Jim-Ray Chen, Chia-Chun Hsu, Guor Mour Her

**Affiliations:** 1 Department of Bioscience and Biotechnology, National Taiwan Ocean University, Keelung 202, Taiwan; 2 Institute of Biopharmaceutical Sciences, National Yang Ming Chiao Tung University, Taipei 112, Taiwan; 3 Institute of Biopharmaceutical Sciences, National Yang-Ming University, Taipei 112, Taiwan; 4 Division of Hemato-Oncology, Department of Internal Medicine, Chang Gung Memorial Hospital, Keelung 204, Taiwan; 5 Department of Pathology, Chang Gung Memorial Hospital, Keelung 204, Taiwan; 6 Department of Radiology, Buddhist Tzu Chi General Hospital, Taichung Branch, Taichung 427, Taiwan; 7 School of Medicine, Tzu Chi University, Hualien 970, Taiwan

The *Cancers* Editorial Office retracts the article, “MicroRNA-21 Plays Multiple Oncometabolic Roles in the Process of NAFLD-Related Hepatocellular Carcinoma via PI3K/AKT, TGF-β, and STAT3 Signaling” [1], as cited above.

Following publication, concerns were brought to the attention of the publisher regarding overlapping panels contained within this article [1]. 

Adhering to our complaint procedure, an investigation was conducted by the Editorial Board and the Editorial Office that confirmed an overlap of panels contained in Figures 5D and 6C,D, representing different experimental conditions. While the authors proactively cooperated with the investigation, the authors were unable to satisfactorily explain the overlapping elements within the above-mentioned figures, nor could they provide raw material to validate these findings. As a result, the Editorial Board and Editor-in-Chief were unable to confirm the reliability of the findings and subsequently decided to retract this article [1], as per MDPI’s retraction policy (https://www.mdpi.com/ethics#_bookmark30, accessed on 8 April 2024) and in line with the Committee on Publication Ethics retraction guidelines (https://publicationethics.org/retraction-guidelines, accessed on 8 April 2024).

This retraction was approved by the Editor-in-Chief of *Cancers*.

The authors agree with this retraction.
